# PTCOG Head and Neck Subcommittee Consensus Guidelines on Particle Therapy for the Management of Head and Neck Tumors

**DOI:** 10.14338/IJPT-20-00071.1

**Published:** 2021-06-25

**Authors:** Alexander Lin, John H. C. Chang, Ryan S. Grover, Frank J. P. Hoebers, Upendra Parvathaneni, Samir H. Patel, Juliette Thariat, David J. Thomson, Johannes A. Langendijk, Steven J. Frank

**Affiliations:** 1 University of Pennsylvania, Philadelphia, PA, USA; 2 Oklahoma Proton Center, Oklahoma City, OK, USA; 3 University of California-San Diego, San Diego, CA, USA; 4 Department of Radiation Oncology (MAASTRO), GROW – School for Oncology and Developmental Biology, Maastricht University Medical Center, Maastricht, The Netherlands; 5 University of Washington, Seattle, WA, USA; 6 Mayo Clinic, Scottsdale, AZ, USA; 7 Radiation Oncology Department, François Baclesse Center/ARCHADE, Normandy University, Caen, France; 8 The Christie NHS Foundation Trust, University of Manchester, Manchester Academic Health Sciences Centre, Manchester, United Kingdom; 9 Department of Radiation Oncology, University of Groningen, University Medical Center Groningen, Groningen, The Netherlands; 10 The University of Texas MD Anderson Cancer Center, Houston, TX, USA

**Keywords:** head and neck cancer, proton therapy

## Abstract

**Purpose:**

Radiation therapy is a standard modality in the treatment for cancers of the head and neck, but is associated with significant short- and long-term side effects. Proton therapy, with its unique physical characteristics, can deliver less dose to normal tissues, resulting in fewer side effects. Proton therapy is currently being used for the treatment of head and neck cancer, with increasing clinical evidence supporting its use. However, barriers to wider adoption include access, cost, and the need for higher-level evidence.

**Methods:**

The clinical evidence for the use of proton therapy in the treatment of head and neck cancer are reviewed here, including indications, advantages, and challenges.

**Results:**

The Particle Therapy Cooperative Group Head and Neck Subcommittee task group provides consensus guidelines for the use of proton therapy for head and neck cancer.

**Conclusion:**

This report can be used as a guide for clinical use, to understand clinical trials, and to inform future research efforts.

## Introduction

External beam radiation therapy is a well-established treatment modality for cancers of the head and neck. It can be used effectively as a single modality for early-stage cancers [1, 2] and for advanced-stage disease, in conjunction with chemotherapy for an organ-preserving approach [3–5], or postoperatively, to improve local regional control and overall survival [6–9]. However owing to exposure of normal tissues, radiation therapy to the head and neck region can be morbid and associated with severe acute and late toxicities [10–16], which can negatively impact posttreatment quality of life [12]. This is a concern, especially given the prevalence of human papillomavirus–associated oropharynx cancer [17, 18] affecting relatively young and healthy patients, and for whom excellent long-term disease outcomes are common [19, 20]. For these patients, form, function, and quality of life long after completion of therapy and cure are of paramount importance.

The unique physical characteristics of a proton beam, with its ability to deliver the majority of radiation therapy over a finite area via the Bragg peak effect, results in avoiding unnecessary radiation exposure to normal tissues, and therefore, may lead to less treatment-associated morbidity. It is for this reason that proton therapy holds promise. However, despite the clear physical and dosimetric advantages of proton therapy, robust clinical data have lagged. The reasons for this are multifactorial, and will be discussed in further detail.

This consensus guideline represents an experts' opinion from The Particle Therapy Co-operative Group (PTCOG) Head and Neck Cancer Subcommittee. This guideline will discuss challenges and barriers to implementation, report supporting evidence by disease subsite, and summarize current and future efforts and recommendations on the treatment of head and neck cancer with proton therapy.

## Challenges and Barriers to Implementation

A significant barrier to widespread implementation and acceptance of proton therapy is the high upfront cost of investment and financing to purchase, construct, and install, and operate a proton beam therapy center [21]. Even with a move to construct less expensive, single room centers there is significant financial risk associated with such a significant investment [22]. Additionally, the higher treatment cost and reimbursement associated with proton therapy, accompanied by an absence of highest-level evidence [23–25] has further hindered wider adoption. For these reasons, even though there are a growing number of proton therapy centers operating worldwide, the availability of proton therapy is still limited and a current barrier to access.

There is growing evidence suggesting that proton therapy may be cost-effective for treatment of high-risk head and neck cancer [26]. Significant reduction of long-term morbidity with proton therapy in younger patients with oropharynx cancer [27] may benefit patients and society at large, with decrease in long-term health care costs (to address treatment-related complications) and higher rates of return to work and productivity. Nonetheless, most payors in the United States still do not reimburse for proton therapy [28]. Insurance approval has been identified as one of the most significant barriers to patient access for proton therapy [29], requiring increased utilization of resources, including valuable clinician time to attempt to obtain approval [29], and often resulting in significant delays to initiation of patient care [29, 30].

There is general agreement that randomized trials are needed to best determine the optimal use of proton therapy for different indications. Meanwhile, alternative methods to evaluate proton therapy have been developed, such as the model-based approach [31], and are being used routinely in countries such as the Netherlands, where patients are selected to receive proton therapy based on anticipated reduction in the probability of treatment-related complications from proton therapy (versus intensity-modulated radiation therapy [IMRT]). Agencies such as the National Cancer Institute and the Patient-Centered Outcomes Research Institute, have dedicated significant resources and funding in support of multiple randomized trials examining proton therapy versus IMRT. However, enrollment in these trials has been hindered by insurance denial of proton therapy for patients enrolled in a study as well as unwillingness to enroll owing to lack of equipoise amongst patients and/or providers [32]. Efforts to improve access, encourage enrollment, and improve insurance coverage of patients participating in these important randomized trials are critical if we are to determine the optimal uses of proton therapy.

## Nasopharynx Cancer

Advances in radiation therapy, such as with IMRT [33, 34] and systemic therapy (such as with induction chemotherapy) [35], have resulted in favorable outcomes for locoregionally advanced, nonmetastatic nasopharynx cancer, with high rates of local regional and systemic control, and long-term survival. However, these modern IMRT series report > grade 3 acute toxicities of 24% to 41% [33, 36] and > grade 3 late toxicities of 12% to 15% [33, 34, 36]. Therefore, the use of proton therapy to improve normal organ sparing and improve acute and late morbidity, while maintaining favorable disease outcomes, is of significant interest and potential application.

Initial studies for nasopharyngeal proton radiation focused on planning comparisons and model-based predictions of toxicity. Widesott et al [37] compared intensity-modulated proton therapy (IMPT) with helical tomotherapy in 6 patients, and found equivalent target coverage and dose homogeneity, but with significant sparing of normal structures such as parotid glands, esophagus, and larynx, with decreased normal tissue complication probability for the parotid glands with IMPT. Taheri-Kadkhoda et al [38] compared IMPT with IMRT in 8 patients, reporting equivalent mean dose delivered to targets between both techniques, but with improved tumor coverage and conformality with IMPT, as well as significant reductions in mean dose to several organs at risk (OARs) with IMPT.

Currently, there are nonrandomized comparative data of proton therapy versus IMRT for nasopharynx cancer. McDonald et al [39] evaluated acute toxicity in a cohort of 40 patients with either cancers of the nasopharynx or paranasal sinus, comparing 3D conformal proton radiation (uniform scanning) to IMRT. Compared with patients who received IMRT, those who received proton therapy were found to have improved sparing of critical structures, as well as corresponding lower rates of opioid requirement for pain at the end of RT, and lower rates of gastrostomy tube dependence at the end of RT and at 3 months post treatment. Holliday et al [40] reported a matched case-control study of 20 patients treated with IMPT, matched to 10 patients treated with IMRT. Those receiving IMPT had significantly lower rates of gastrostomy tube (G-tube) insertion (20% versus 65%), with a reduction in mean oral cavity dose to less than 26 Gy from proton therapy, associated with decreased G-tube placement.

One currently enrolling randomized trial is comparing photon to proton radiation therapy for nasopharynx cancer. The Shanghai Proton and Heavy Ion Center is conducting a randomized phase II study of photon versus proton radiation therapy for patients with newly diagnosed disease (NCT04528394) [41]. With a planned total enrollment of 136, patients in both arms will receive a boost via carbon-ion after receiving an initial 56 Gy over 28 fractions. The primary outcome of the trial is the rate of > grade 2 xerostomia 6 months after treatment.

Given the complex anatomic location of the nasopharynx located close to critical organs (such as the optic structures, brainstem, cochlea, temporal lobes), and the extent of the target volumes required to cover the extent of disease, the most advanced forms of proton treatment planning and delivery are recommended. Robustly optimized, multifield IMPT is superior to single-field optimization [42], and recommended. Additionally, given potential daily variation in anatomy at the skull base, as well as anatomic change secondary to disease response, high-quality daily anatomic imaging (such as with cone-beam computed tomography) is needed for quality assurance and to determine if/when plan adaptation is required [43].

In summary, given the morbidity commonly seen with treatment of nasopharynx cancer with the most advanced, non–proton radiation techniques, proton therapy can be used to improve normal tissue sparing and therefore decrease toxicity. Current efforts, such as the randomized trial comparing IMPT with IMRT, and others focused on longitudinal data collection and reporting of patient-reported outcomes, will continue to provide evidence on the potential impact of proton therapy to improve the therapeutic ratio and outcomes for patients with nasopharynx cancer.

## Reirradiation

Advancement and intensification of multimodality therapy for head and neck malignancies have improved outcomes. However, local regional failures remain a significant risk at approximately 15% to 30% in large series from the European Organisation for Research and Treatment of Cancer (EORTC) and Radiation Therapy Oncology Group (RTOG). In addition, the risk of second malignant neoplasms in the head and neck after prior therapies for head and neck cancer is also on the order of approximately 1% to 5% per year.

Options for definitive therapy are usually limited. Surgery can be performed only for nonextensive recurrences and is often associated with high complication/morbidity rates and need for further adjuvant therapy. Chemotherapy or immunotherapy alone can provide some palliation and disease control, but ultimately it is not generally curative. Recently, immunotherapy has shown some promise; however, only a minority of patients derive long-term benefit.

Reirradiation is often necessary as an adjuvant after surgery or as the definitive modality to treat local regional recurrences or a second malignancy. The aims of treatment are 2-fold: attempt at cure and gain local control of disease. Both aims are important in view of the complications associated with local disease progression including problems with swallowing, bleeding, breathing, pain, and aspiration. Data from large cooperative groups and single institutions have demonstrated efficacy for reirradiation in this setting [44, 45].

While reirradiation can be the only curative option, the proximity to previously irradiated critical OARs can prevent delivery of a potentially curative radiation dose. The advantaged physical properties of particle therapy may (1) improve target volume coverage and thereby tumor control, and (2) decrease the integral dose and thereby reduce severe early and late toxicities. However, this requires prospective evaluation, ideally within a clinical trial setting. A recently published series [46] reported clinical results of proton reirradiation in 17 patients with recurrent nasopharynx cancer. A median dose of 60 GyRBE was delivered, with no reported > grade 3 acute toxicity, and a 23.5% rate of > grade 3 late toxicity and 1 patient with a fatal carotid blowout. At 18 months, overall survival and local control were 54.4% and 66.6%, respectively. Other recent publications from MD Anderson Cancer Center (MDACC), Indiana University Health, Memorial Sloan Kettering Cancer Center (MSKCC), and Northwestern Medicine Chicago Proton Center have demonstrated preliminary data with a median follow-up of 1 to 2 years [47–49]. Local regional control was 70% to 80% with overall survival rates of 65% to 80% at 12 months [47, 49], and approximately 35% at 2 years [48]. Grade 3+ acute and late toxicities ranged from 12% to 30% including approximately 2% to 5% treatment-related mortality seen mainly from carotid hemorrhage. Long-term feeding tube dependence was approximately 20% to 25%. These results compare favorably to historical controls from the RTOG and University of Chicago where overall survival was below 30%, grade 5 toxicities were 10% or higher, and feeding tube dependence was above 50% with photons. An in silico trial and dosimetric studies showed reduction of mean dose to most OARs, assessed with IMPT versus IMRT [50, 51].

There are several prospective studies that are currently open and actively accruing for proton reirradiation. An MSKCC phase II study (NCT03217188) is comparing conventionally fractionated full-dose proton reirradiation (70 Gy in 2 Gy fractions) versus hypofractionated palliative reirradiation (3.7 Gy bid × 2 days, followed by a 4-week break, repeated up to 4 cycles), with a primary outcome of 1-year local regional control [52]. An MDACC phase II study (NCT03164460) is comparing stereotactic photon radiation therapy versus conventionally fractionated proton therapy, with a primary outcome measure of comparing 2-year rates of grade 3 or higher toxicity between the 2 arms [53]. Given the emergence of immunotherapy in the treatment of recurrent or metastatic disease, the Mayo Clinic is investigating the role of proton stereotactic radiation therapy plus immunotherapy (nivolumab) for recurrent/progressive local regional or metastatic head and neck cancer (NCT03539198) [54].

Caution must be exercised whenever reirradiation is to be considered. Careful patient evaluation to assess performance status, morbidity from prior RT, and details of the prior RT course (time since treatment, dose, areas treated) are critical to determine whether additional RT can and should be given. For situations where a disease recurrence abuts or involves a critical organ, the risks of severe toxicity with reirradiation are not appreciably different regardless of RT modality (IMRT versus proton). In these cases, or for others where the risks associated with any type of reirradiation are appreciably less than the potential benefits, the decision to recommend against further radiation therapy is as important as the decision to recommend it in situations where it is warranted.

While still a daunting prospect, reirradiation for recurrent or new cancers of the head and neck is necessary for many patients. Utilization of multiple treatment strategies is possible. Particle therapy may provide the least invasive and most effective approach in this setting or in combination with surgical salvage and/or systemic therapy. Decisions to offer reirradiation need to be made on an individual basis between patient and provider, after consultation and consideration of its inherent risks versus benefits.

## Sinonasal

Cancers of the sinonasal region frequently require multidisciplinary management with surgery, radiation, and/or chemotherapy. Delivery of safe and adequate radiation therapy is difficult owing to the anatomic constraints posed by adjacent critical structures such as the brain, brainstem, and optic structures.

In a systematic review and meta-analysis of 41 observational studies [55], subgroup analysis showed the use of proton beam therapy, compared with IMRT, for paranasal sinus and nasal cavity cancers improved disease-free survival at 5 years (relative risk, 1.44; 95% confidence interval [CI], 1.01-2.05; *P* = .045) and local regional control at longest follow-up (relative risk, 1.26; 95% CI, 1.05-1.51; *P* = .011). However, it should be noted that this review also found that charged particle therapy was associated with a greater risk of neurologic toxicity than photon therapy. More recent retrospective studies of proton beam therapy for sinonasal cancers continue to report encouraging local regional control rates [56, 57]. In the study from Dagan et al [56], 84 patients received primary (13%) or postoperative (87%) proton beam therapy with an overall 3-year local control rare of 83%; and in the 64 of 73 cases where gross total surgical resection was achieved, the 3-year local control rate was 90%.

Given the promising results that have been reported, proton therapy should be considered whenever possible for patients requiring radiation therapy. Similar to the nasopharynx, with target volumes located near critical organs, treatment with robust-optimization, multifield IMPT, coupled with daily anatomic imaging to inform the need for treatment adaptation, is recommended to achieve the best results and to reduce the risk of long-term neurotoxicity. Given the relative uncommon nature of this diagnosis, coupled with the heterogeneity in the histology and location of cancers affecting this area, it is unlikely that a large-scale, multicenter randomized study will be performed comparing IMRT to IMPT in this setting; however, continued long-term data collection and reporting on patient outcomes, along with anticipated results from current randomized trials for other head and neck cancer subsites, will be used to inform and guide future recommendation on the use of proton therapy in this setting.

## Postoperative

Postoperative proton beam therapy is used in situations where compared with IMRT, there are dosimetric benefits (improved target volume coverage, or a reduction in dose to OARs) that translate into improved local tumor control or reduced treatment-related toxicities (eg, ipsilateral treatment of salivary gland cancers, and treatment of oropharyngeal cancers).

Romesser et al [58] compared IMRT and proton beam therapy for the ipsilateral treatment of salivary gland cancers. For 41 consecutive patients, 37 of 41 received postoperative ipsilateral radiation using IMRT (23 of 41) or proton beam therapy (18 of 41). There was similar target volume coverage between modalities, but IMRT compared with proton beam therapy plans had significantly higher median maximum doses to the brainstem (29.7 Gy versus 0.6 GyRBE, *P* < .001), spinal cord (36.3 Gy versus 1.9 GyRBE, *P* < .001), and mean oral cavity (20.6 Gy versus 0.94 GyRBE, *P* < .001). For proton beam therapy, this corresponded to lower rates of grade 2 or worse acute dysgeusia (5.6% versus 65.2%, *P* < .001), mucositis (16.7% versus 52.2%, *P* = .019), and nausea (11.1% versus 56.5%, *P* = .003). While the data were retrospective, these differences in clinically meaningful end points are of a magnitude of 3 to 10 times.

In a planning comparison study of proton beam therapy and IMRT following transoral surgery for oropharyngeal cancer, where both primary site and bilateral neck were included as target volumes, IMRT plans had significantly higher mean doses to the oral cavity (17.7 Gy versus 2.9 GyRBE, *P* < .001), contralateral parotid gland (18.0 Gy versus 13.6 GyRBE, *P* < .001), and contralateral submandibular gland (36.1 Gy versus 32.5 GyRBE, *P* = .03) [59].

A currently open and accruing clinical study at MSKCC is a phase II randomized study of proton versus photon beam RT in the treatment of unilateral head and neck cancer (NCT029235870). This study seeks to randomly assign 132 patients who require unilateral neck RT to receive either IMRT or proton therapy to a dose of 60 to 66 Gy, with a primary outcome focused on number of patients with grade 2 or greater acute mucositis [60].

As for clinical results, in the setting of oropharynx cancer, Sharma et al [61] reported on patient-reported quality of life outcomes in patients undergoing postoperative oropharyngeal radiation with proton therapy versus IMRT. Of a total of 64 patients (33 receiving volumetric modulated arc therapy versus 31 receiving proton therapy), proton therapy resulted in significantly lower doses to normal organs, particularly doses to salivary glands and oral cavity, with higher scores in head and neck specific, as well as general quality of life. The most significant improvement in quality of life metrics seen with proton therapy 6 months after completion of RT was for xerostomia and appetite changes. Prospective studies completed at the University of Pennsylvania (NCT02159703) [62] and the Mayo Clinic (NCT02736786) [63], examining the use of mucosal-sparing proton beam therapy to the necks alone following transoral surgery for oropharyngeal cancer, have completed accrual. Mucosal-sparing proton therapy allows for dramatic dose reduction to the midline structures such as the oral cavity and pharyngeal axis, with proton therapy delivering significantly better sparing than IMRT to the mucosa of the resected primary tumor bed [64].

In summary, the use of proton therapy for adjuvant radiation, even in situations where bilateral neck radiation is not required, could improve patient outcomes with respect to toxicity and quality of life. Therefore, its role in the postoperative setting is promising and should be considered.

## Oropharynx

Oropharynx cancer, given excellent disease outcomes and long-term patient survival but high levels of late toxicities with IMRT, is an ideal indication for consideration of proton therapy for toxicity mitigation. It is a model on which new techniques of proton radiation can be tested and implemented. For example, the concept of robust optimization with multifield optimized (MFO) IMPT, which allows for the greatest potential benefit of target coverage and organ sparing with pencil-beam scanning proton therapy, was first tested in the setting of oropharynx cancer [65]. MFO was compared with single-field optimization and found to show improved clinical target volume coverage and homogeneity, while simultaneously improving sparing of critical structures such as the pharyngeal constrictors and the larynx [42]. Robust MFO optimization has now been implemented into clinical systems and is being used for proton treatment planning and treatment delivery.

From a clinical standpoint, as mentioned above, proton therapy for oropharynx in the postoperative setting appears to be superior to IMRT, with gains in patient-reported outcome and quality of life [61]. A case matched analysis of 150 patients with oropharynx cancer (50 treated with IMPT versus 100 treated with IMRT) from the MDACC examined clinical outcomes of the 2 modalities [66]. While there were no differences in overall survival between the 2 modalities, patients receiving IMPT were far less likely to have grade 3 weight loss or presence of G-tube at 3 months (odds ratio [OR] = 0.44; 95% CI: 0.19-1.0) or 1 year after treatment (OR = 0.23; 95% CI: 0.07-0.73). Prospective, multicenter randomized trials of IMPT versus IMRT for oropharynx are currently underway in the United States and the United Kingdom. The US multicenter trial, being led by MDACC (NCT01893307), is a phase III study of 440 patients in which patients with locoregionally advanced oropharynx cancer will receive organ-preservation chemoradiation, with RT randomized between IMRT and IMPT. The primary outcome of the trial is comparison of 3-year progression-free survival between the 2 techniques, with a secondary outcome examining factors such as patient-reported outcomes, physician-reported toxicity, quality of life, and cost-benefit economic analyses, measure of rates and severity of late grade 3 to 5 toxicity [67]. The National Health System in England has recently launched their first prospective, randomized clinical trial for proton therapy (TORPEdO, TOxicity Reduction using Proton bEam therapy for Oropharyngeal cancer). It is a phase III, multicenter, randomized controlled study for patients with oropharyngeal cancer requiring definitive, organ-preserving chemoradiation and bilateral neck treatment. Patients are randomly assigned to IMRT versus IMPT, with primary outcome of late treatment-related toxicity.

In summary, given the importance of chronic toxicity mitigation for expected long-term survivors of oropharynx cancer, proton therapy should be considered when radiation therapy is indicated (either as single modality, in combination with chemotherapy for organ preservation, or in an adjuvant setting). Participation in clinical trials, such as those mentioned above, is strongly encouraged. When trial participation is not feasible, treatment with proton therapy, whenever possible, is recommended, given the existing (nonrandomized) data suggesting improved therapeutic ratio.

## Future Directions and Conclusions

Proton therapy is an established and safe modality for the treatment of patients with head and neck cancers. The superior dosimetric conformity and organ-sparing capabilities appear to correspond with improved patient outcomes when compared with IMRT per the existing literature, which suggests that proton therapy may ultimately prove to be the more cost-effective modality. Table 1 summarizes relevant findings and recommendations by disease subsite/indication. Confirmatory evidence needs to be collected and published, in order to address the current barriers limiting patient access. In cases where normal organ constraints cannot be met with IMRT, consideration of proton therapy is justifiable [68]. Participation in clinical trials (Table 2), particularly in phase III randomized trials comparing proton therapy to IMRT, as described above, is strongly encouraged. When clinical trial participation is not feasible, predictive tools, like the model-based predictive approach [69], may allow clinicians to identify patients most likely to derive benefit from proton therapy in settings where its availability is limited.

**Table 1.** Relevant findings and recommendations, by subsite/indication.

**Subsite/ indication**	**Relevant findings**	**Recommendation**
Nasopharynx	Nonrandomized, comparative data showing less toxicity with proton therapy.	Consider proton therapy whenever feasible. Most advanced treatment, imaging, and adaptation techniques should be used to minimize risk of neurotoxicity, given anatomic location.
Reirradiation	Local regional and toxicity with proton therapy favorable when compared to historical controls. Clinical trials directly comparing proton therapy to IMRT currently enrolling.	Careful evaluation required for each patient to determine risks/benefits of reirradiation. Enrollment in clinical trial encouraged whenever possible.
Sinonasal	Systematic review/meta-analysis showing improved local regional control and disease-free survival with proton therapy over IMRT, but with greater risk of neurotoxicity.	Consider proton therapy whenever feasible. Most advanced treatment, imaging, and adaptation techniques should be used to minimize risk of neurotoxicity, given anatomic location.
Postoperative	Nonrandomized, comparative data showing less toxicity and improved patient-reported outcomes with proton therapy. Clinical trials directly comparing proton therapy to IMRT currently enrolling.	Consider proton therapy whenever feasible. Enrollment in clinical trial encouraged whenever possible.
Oropharynx	Nonrandomized, comparative data showing less toxicity and improved patient-reported outcomes with proton therapy. Model-based methods being used to select patients most appropriate for proton therapy. Clinical trials directly comparing proton therapy to IMRT currently enrolling.	Consider proton therapy whenever feasible. Enrollment in clinical trial encouraged whenever possible.

**Abbreviation:** IMRT, intensity-modulated radiation therapy.

**Table 2.** Current clinical trials examining the use of proton therapy in the treatment of head and neck cancer.

**Subsite or indication**	**Title**	**ClinicalTrials.gov identifier**	**Phase**	**Center**	**End Point**
Nasopharynx	Proton Versus Photon Radiotherapy for Nasopharyngeal Carcinoma	NCT04528394	II (randomized)	Shanghai Proton and Heavy Ion Center	Rate of > grade 2 xerostomia 6 months after treatment
Reirradiation	Proton Re-Irradiation for Recurrent Head and Neck Cancer	NCT03217188	II (nonrandomized)	Memorial Sloan Kettering Cancer Center	Local regional control (12 mo)
Reirradiation	Stereotactic Body Radiation Therapy or Intensity Modulated Radiation/Proton Therapy in Treating Patients With Recurrent Head and Neck Cancer	NCT03164460	II (randomized)	MD Anderson Cancer Center	2-y CTCAE v4.0 > grade 3 toxicity rate
Reirradiation	Study of Proton SBRT and Immunotherapy for Recurrent/Progressive Locoregional or Metastatic Head and Neck Cancer	NCT03539198	Observational	Mayo Clinic	Objective response rate
Postoperative	Study of Proton Versus Photon Beam Radiotherapy	NCT02923570	II (randomized)	Memorial Sloan Kettering Cancer Center	Rate of > grade 2 acute mucositis
Oropharynx	Randomized Trial of Intensity-Modulated Proton Beam Therapy (IMPT) Versus Intensity-Modulated Photon Therapy (IMRT) for the Treatment of Oropharyngeal Cancer of the Head and Neck	NCT01893307	III (randomized)	Mulitcenter (sponsor: MD Anderson Cancer Center)	3-y progression-free survival
Oropharynx	TORPEdO		III (randomized)	National Health System (UK)	Rates of late treatment-related toxicity between IMRT and IMPT

**Abbreviations:** CTCAE v4.0, Common Terminology Criteria for Adverse Events, version 4.0; IMRT, intensity-modulated radiation therapy; IMPT, intensity-modulated proton therapy.
